# A Systems Nephrology Approach to Diabetic Kidney Disease Research and Practice

**DOI:** 10.1159/000531823

**Published:** 2023-09-11

**Authors:** William P. Martin, Neil G. Docherty

**Affiliations:** a Diabetes Complications Research Centre, School of Medicine, Conway Institute of Biomolecular and Biomedical Research, University College Dublin, Belfield, Dublin D04 V1W8, Ireland

**Keywords:** systems nephrology, systems biology, personalised medicine, diabetic kidney disease, diabetic nephropathy, omics, machine learning, deep learning

## Abstract

**Background:**

Diagnosis and staging of diabetic kidney disease (DKD) via the serial assessment of routine laboratory indices lacks the granularity required to resolve the heterogenous disease mechanisms driving progression in the individual patient. A systems nephrology approach may help to resolve mechanisms underlying this clinically apparent heterogeneity, paving a way for targeted treatment of DKD.

**Summary:**

Given the limited access to kidney tissue in routine clinical care of patients with DKD, data derived from renal tissue in preclinical model systems, including animal and *in vitro* models, can play a central role in the development a targeted systems-based approach to DKD. Multi-centre prospective cohort studies, including the Kidney Medicine Precision Project (KPMP) and the European Nephrectomy Biobank (ENBiBA) project, will improve access to human diabetic kidney tissue for research purposes. Integration of diverse data domains from such initiatives including clinical phenotypic data, renal and retinal imaging biomarkers, histopathological and ultrastructural data, and an array of molecular omics (transcriptomics, proteomics, etc.) alongside multi-dimensional data from preclinical modelling offers exciting opportunities to unravel individual-level mechanisms underlying progressive DKD. The application of machine and deep learning approaches may particularly enhance insights derived from imaging and histopathological/ultrastructural data domains.

**Key Messages:**

Integration of data from multiple model systems (*in vitro*, animal models, and patients) and from diverse domains (clinical phenotypic, imaging, histopathological/ultrastructural, and molecular omics) offers potential to create a precision medicine approach to DKD care wherein the right treatments are offered to the right patients at the right time.

## The promise of personalised diabetic kidney disease care

Diabetic kidney disease (DKD) develops in approximately 40% of people with type 2 diabetes mellitus and is the leading cause of end-stage kidney disease worldwide [1]. The majority of excess cardiovascular and all-cause mortality attributable to diabetes mellitus occurs in those with kidney disease [2]. There is a significant residual risk of progressive renal functional decline despite treatment with the current backbone of renin-angiotensin-aldosterone system (RAAS) blockade and sodium-glucose cotransporter-2 inhibitors (SGLT2is) [3, 4]. Glucagon-like peptide-1 receptor agonists (GLP1RAs) including liraglutide and semaglutide [5], the dual GIP and GLP-1 receptor agonist tirzepatide [6], and the nonsteroidal mineralocorticoid receptor antagonist finerenone [7] have also been shown to improve cardiovascular and renal outcomes in people with DKD recently. Intentional weight loss approaches, including metabolic surgery, may also have a role in slowing DKD progression [8, 9]. However, outcomes for people with DKD remain suboptimal and new treatment approaches are needed.

DKD is a multifactorial disease, with substantial heterogeneity observed in its pathophysiology and treatment-responsiveness at the individual level [10]. While genetic factors contribute to the development of DKD, these interact with, and are modified by, multiple exogenous mediators such as environmental or dietary factors, leading to a weak hereditability pattern [10]. Critical determinants of renal functional reserve including nephron endowment, age-associated decline in nephron number, acute kidney injury (AKI) history, and the intrinsic capacity of nephrons to adapt to haemodynamic and metabolic stressors vary widely amongst people with type 2 diabetes [11]. Furthermore, while disease-modifying treatments for DKD exist, such as RAAS blockade and SGLT2is, the prognosis remains poor because not all patients respond optimally to these treatments [10].

Routine management of DKD relies on serial assessment of a limited number of biochemical parameters, such as estimated glomerular filtration rate (eGFR) derived from serum creatinine and the urinary albumin-to-creatinine ratio (uACR), to diagnose and stage the disease [12]. Patients are staged according to KDIGO guidelines by albuminuria (A1-A3) and eGFR (G1-G5) categories, with increasing albuminuria (A3 being the highest level of albuminuria) and declining eGFR (G5 being the lowest eGFR category) reflective of advancing DKD [12]. Both eGFR and uACR have particular limitations in the identification of early DKD [12]. Furthermore, many patients with DKD are recognised to experience progressive loss of eGFR and premature mortality in the absence of established albuminuria [13]. While kidney histopathology may help to diagnose and prognosticate DKD, kidney biopsies are not routinely pursued in patients with diabetes unless a chronic kidney disease (CKD) aetiology other than DKD is suspected [14]. As well as the inherent limitations of eGFR and uACR, patients with DKD of a similar KDIGO disease stage may be misclassified as having a similar phenotype despite heterogeneous disease mechanisms driving disease progression at the individual level [15].

Rather than focusing on one pathophysiologic mechanism at a time, a systems biology approach to DKD incorporating a wide spectrum of information including clinical phenotypic data, histopathologic data, imaging biomarkers, and molecular multi-omics data promises to provide a more complete understanding of the interacting disease mechanisms driving disease progression in a given patient [16]. A central aim of systems nephrology is the identification of molecular mechanisms underpinning progressive DKD, which may enable the classification of DKD based on mechanisms rather than the current system of KDIGO staging according to eGFR and uACR categories [15, 17, 16, 12]. The goal is that patients with DKD can be stratified based on their molecular characteristics, thereby defining relatively homogeneous subgroups of patients wherein patients in each subgroup have a similar prognosis and responsiveness to established therapies [15, 17, 16]. This would also facilitate the conduct of more meaningful and efficient randomised clinical trials (RCTs) of novel therapies, as treatments could be targeted based on disease mechanisms to those most likely to benefit, thereby also preventing exposure to those patients unlikely to respond based on their molecular profile.

As well as identifying diagnostic and prognostic biomarkers of DKD, a systems nephrology approach may facilitate the translation of novel therapies for DKD. This may be achieved through the identification of novel drug targets or indeed by drug repurposing using databases of perturbagen-driven gene expression profiles, such as Connectivity Map [18]. The former approach has led to promising studies of baricitinib, a small molecule inhibitor of JAK1 and JAK2, in DKD [19] while the latter approach recently identified lysine deacetylase inhibition as a potential treatment option for progressive proteinuric CKD [20]. Similarly, we combined *in silico* deconvolution and network pharmacology approaches to identify PPARα-stimulated proximal tubular fatty acid oxidation as a key mechanism underpinning the renoprotective effects of intentional weight loss plus multi-modal pharmacotherapy in animals models of DKD [21, 22], highlighting the potential for fibrate therapy to synergise with intentional weight loss to mitigate DKD progression.

## Model systems and data sources

**Fig. 1:**
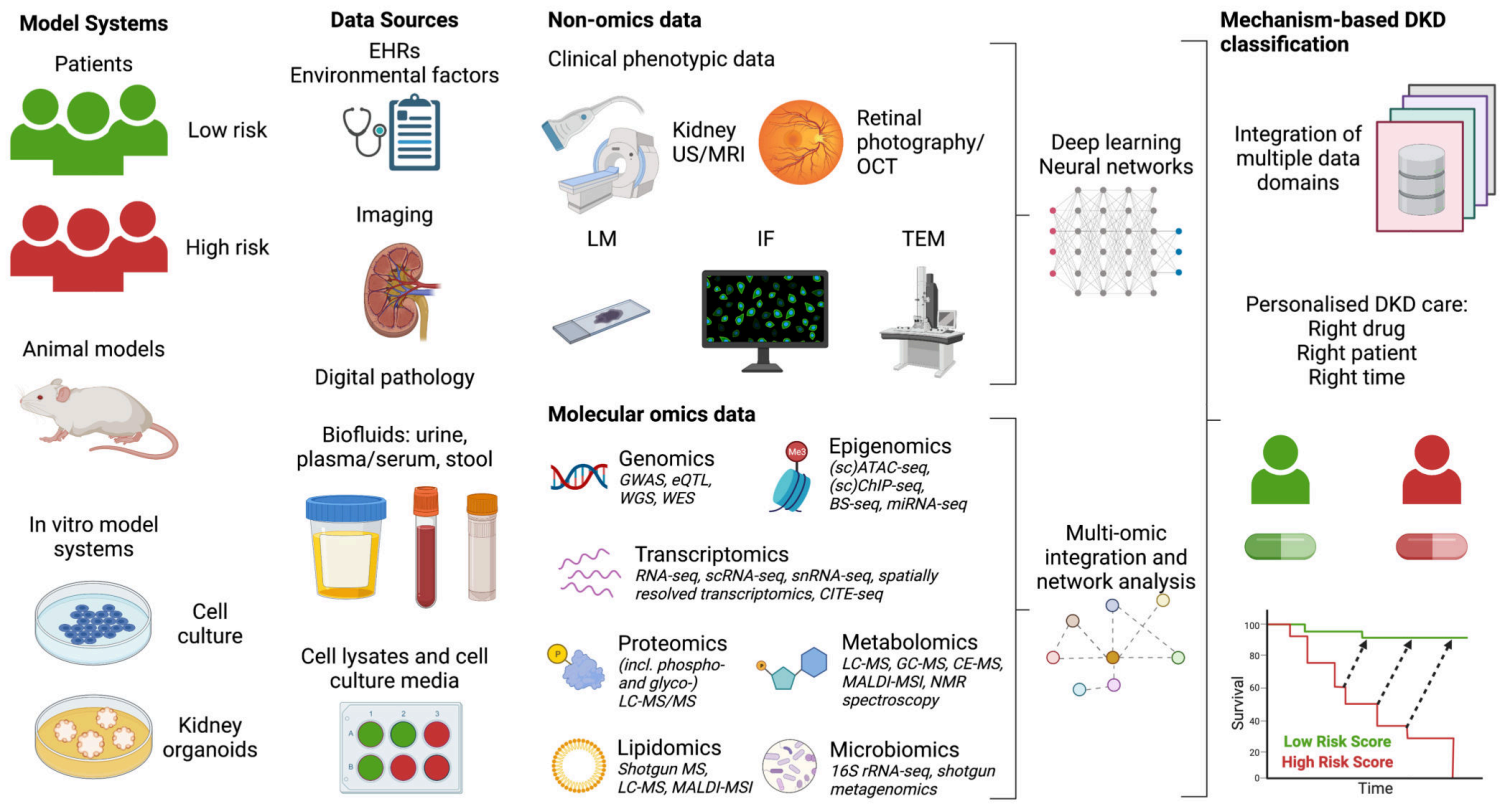
Overview of a comprehensive systems nephrology approach applied to DKD research. Created with BioRender.com. 16S rRNA-seq, 16S ribosomal RNA sequencing; ATAC-seq, assay for transposase-accessible chromatin using sequencing; BS-seq, bisulfite sequencing; CE-MS, capillary electrophoresis–mass spectrometry; ChIP-seq, chromatin immunoprecipitation followed by sequencing; CITE-seq, cellular indexing of transcriptomes and epitopes by sequencing; DKD, diabetic kidney disease; EHR, electronic health record; eQTL, expression quantitative trait loci; GC-MS, gas chromatography–mass spectrometry; GWAS, genome-wide association study; IF, immunofluorescence; LC-MS, liquid chromatography–mass spectrometry; LM, light microscopy; MALDI-MSI, matrix assisted laser desorption/ionization mass spectrometry imaging; miRNA-seq, microRNA sequencing; MRI, magnetic resonance imaging; MS, mass spectrometry; NMR, nuclear magnetic resonance; OCT, optical coherence tomography; RNA-seq, ribonucleic acid sequencing; scRNA-seq, single-cell RNA sequencing; snRNA-seq, single-nucleus RNA sequencing; TEM, transmission electron microscopy; US, ultrasound; WES, whole exome sequencing; WGS, whole genome sequencing.

An overview of a comprehensive systems nephrology approach to DKD research is outlined in Figure 1. Elements from this which are considered essential to the delivery of precision medicine for people with DKD are further expanded upon in Table 1, outlining the rationale for, and caveats of, deploying individual elements of the systems nephrology paradigm.

## Animal and human studies

As outlined in Figure 1, studies across multiple model systems are necessary for a comprehensive systems nephrology approach to advance DKD care. As exemplified by the implication of JAK-STAT pathway activation in DKD pathogenesis, integrative analyses of data derived from cross-species studies may be particularly informative [17]. As animal models do not reliably recapitulate all features of human DKD [23], access to human kidney tissue from patients with DKD and from healthy controls is essential. In many respects, the delivery of routine nephrology clinical care is uniquely positioned to be informed by systems-level analysis [15]. The availability of kidney tissue from biopsies and urine samples provides an opportunity to leverage insights from systems biology to improve patient care. In the context of DKD, however, access to kidney tissue is more limited as kidney biopsies are usually only pursued when a CKD aetiology other than diabetes mellitus is suspected [14]. Thus, molecular studies of kidney tissue from biopsied cohorts of patients with DKD come with the caveat that such cohorts may be over-represented with patients with unusual mechanisms of disease progression or with alternative or additional CKD aetiologies, and may thus fail to identify the most common disease mechanisms [14].

This point underscores the importance of initiatives such as the Kidney Precision Medicine Project (KPMP), which is a multi-centre prospective cohort study of people with CKD and AKI who undergo a protocol kidney biopsy for research purposes at study entry [14]. The KPMP is focusing on the most prevalent kidney diseases, and thus in the context of CKD, is specifically recruiting patients with CKD attributed to diabetes, hypertension, or both [14]. Other multi-centre prospective cohort studies are also being conducted with the aim of improving access to human DKD tissue for research purposes. For example, the Transformative Research in Diabetic Nephropathy (TRIDENT) consortium is coordinating the collection of kidney tissue from patients with DKD undergoing clinically indicated kidney biopsies across multiple centres in the United States [24]. The European Nephrectomy Biobank (ENBiBA) project is a multi-centre initiative of the Diabesity working group of the European Renal Association aiming to collect renal tissue from patients with diabetes, obesity, and metabolic syndrome at the time of nephrectomy for other indications [25].

A limitation of such cohort studies of human diabetic kidney tissue is that biopsies are obtained at a single point in time and thus offer a snapshot into molecular mechanisms underpinning disease progression to that point. Longitudinal access to human kidney tissue for research purposes is limited by the invasive nature of the biopsy procedure, in effect making it very challenging to directly assess histological response to therapeutic interventions in human DKD. This underscores the importance of preclinical studies of therapeutic interventions in DKD [23], as well as the development of alternative means of assessing treatment response in patients with DKD.

In this context, urine samples are readily accessible and proposed as a ‘liquid biopsy’ which may offer insights into the molecular state of the kidney [15]. Other biofluids such as plasma/serum samples and stool specimens are also readily accessible and may offer insights into treatment response. Imaging surrogates of treatment response, which could be obtained non-invasively by routinely available imaging modalities such as ultrasound or magnetic resonance imaging (MRI), are also sought [26]. Early changes in routinely available clinical parameters may also predict longer-term clinical outcomes after an intervention in patients with DKD [10]. For example, the parameter response efficacy (PRE) score, which incorporates data from multiple cardiovascular and renal risk markers, has been developed for this purpose and has been demonstrated to accurately predict treatment response to several drug classes, including angiotensin-II receptor blockers (ARBs), glucagon-like peptide-1 receptor agonists (GLP1RAs), endothelin receptor antagonists, and SGLT2is [10].

## *In vitro* studies

Aside from animal and human studies, *in vitro* studies in primary and immortalised renal cell lines offer a unique opportunity to explore mechanisms underpinning DKD progression. The development of 3D kidney organoids from induced pluripotent stem cells has been a major advance in the field of discovery nephrology research [27, 28]. Kidney organoids recapitulate many aspects of the cellular complexity of the human kidney, and single-cell RNA sequencing (scRNA-seq) technologies can be applied to kidney organoids as a quality control measure to confirm the presence of specific cell types and to ensure reproducibility in the differentiation process [27, 28].

Kidney organoids may offer a powerful means of understanding molecular mechanisms underpinning DKD progression at single-cell resolution. For example, the utility of integrating scRNA-seq data from kidney organoids with bulk RNA-seq data from human glomerular tissue was highlighted by a recent study which demonstrated shared gene expression signatures between glomerular cells in kidney organoids and in the developing human kidney, elements of which were also found to be reactivated in progressive human glomerular disease [29]. Compared with standard 2D cell culture methods, 3D organoids may also offer an opportunity to study responses to genetic or pharmacological therapeutic approaches in multiple kidney cell types simultaneously, thus highlighting cell-specific mechanisms which could be targeted to attenuate CKD progression [17].

However, certain limitations of kidney organoids are recognised [27, 28]. For example, current kidney organoid models lack a dedicated circulation and fenestrated glomerular capillaries [27], primarily due to the paucity of endothelial cells, which are estimated to represent just 0.1 to 0.2% of all kidney organoid cells by single-cell analysis [30]. Furthermore, the tissue culture media used in kidney organoid differentiation protocols are high in glucose, thereby potentially confounding disease versus control comparisons for studies with a DKD focus [27]. It is unclear whether organoids would mature normally in the presence of a normal glucose concentration [27]. Thus, refinements to kidney organoid differentiation protocols will be necessary before they can realise their full potential as a comprehensive *in vitro* model of DKD [27]. Renal slice culture from nephrectomy specimens could offer an additional platform, which although less amenable to genetic manipulation than organoids, have advantages regarding cellular composition and tissue integrity and maturity [31]. Integration of target discovery in organoids with subsequent assessment of pharmacological responses in renal slice culture could offer a pragmatic means of mitigating attrition rate rates between preclinical and early phase clinical studies.

## Non-omics data

### Clinical phenotypic data

Clinical phenotypic data derived from electronic health records (EHRs) are a rich resource which may be harnessed to individualise prognosis and treatment response [15, 16]. Clustering of patients with newly diagnosed adult-onset diabetes on the basis of 6 variables (age, body-mass index (BMI), glycated haemoglobin, glutamic acid decarboxylase antibodies, and homeostatic model assessment 2 (HOMA2) estimates of β-cell function and insulin resistance) across multiple independent Scandinavian cohorts reproducibly identified 5 subgroups of patients with substantially different risks of diabetes complications [32]. In particular, the severe insulin resistant diabetes (SIRD) cluster, characterised by high BMI, hyperinsulinaemia, and mild hyperglycaemia, had the highest risks of incident DKD and end-stage kidney disease (ESKD) [32].

### Prognostication by individual proteins

Assessment of individual proteins may also be used to enhance prognostication of adverse CKD outcomes in patients with diabetes [10]. For example, circulating levels of soluble tumour necrosis factor receptor-1 (sTNFR1) and soluble tumour necrosis factor receptor-2 (sTNFR2) have been demonstrated to independently predict progressive renal functional decline, ESKD, and cardiovascular and all-cause mortality across multiple cohorts of patients with diabetes mellitus [33–36]. More broadly, 17 proteins from the tumour necrosis factor-receptor superfamily, including sTNFR1 and sTNFR2, were strongly associated with 10-year ESKD risk in cohorts of patients with type 1 and type 2 diabetes mellitus [35]. Circulating levels of kidney injury molecule-1 (KIM-1) and N-terminal pro-brain natriuretic peptide (NT-proBNP) also strongly predict DKD progression [37, 38]. The quantification of 11 serum biomarkers by two novel multiplex arrays on a clinical-grade analyser improved prediction of CKD progression and mortality in patients with CKD (25% with diabetes mellitus). sTNFR1, neutrophil gelatinase-associated lipocalin (NGAL), C-reactive protein, and complement 3a with cleaved C-terminal arginine (C3a-desArg) were identified as the most strongly prognostic biomarkers [39].

### Renal and retinal imaging biomarkers

The large amount of data generated by kidney imaging with clinically available modalities such as ultrasound and MRI is a potentially rich source of biomarkers to inform DKD prognostication and treatment response [26, 16]. Such biomarkers may be human-visible and quantifiable by manual, semi- automated, or automated means. One such example in the field of autosomal dominant polycystic kidney disease is total kidney volume (TKV), a surrogate marker of disease progression which correlates with cyst volume and decline in eGFR [40]. An automated segmentation method based on deep learning has been developed to calculate TKV in a fast and reproducible manner, and demonstrated good agreement with TKV values calculated from manual segmentations [41].

Alternatively, in the field of computer vision, high-dimensional numeric data may be extracted from radiologic images and analysed using machine or deep learning approaches to classify images and detect patterns which are not visible to the human eye. As part of the Biomarker Enterprise to Attack Diabetic Kidney Disease (BEAt-DKD) consortium, the prospective, multi-centre iBEAt cohort study is the largest DKD imaging study to date and aims to determine whether ultrasound and MRI renal imaging biomarkers provide insight into the heterogeneity in DKD pathogenesis and can prognosticate adverse outcomes amongst patients with type 2 DKD [26]. A key advantage of imaging over other biomarker approaches to personalise DKD management is the fact that the left and right kidneys as well as the renal cortex and medulla can be assessed independently, potentially providing more granularity into functional and structural heterogeneity amongst patients with DKD [26].

As diabetic retinopathy and DKD are closely intertwined as microvascular complications of diabetes mellitus, retinal imaging is also a potentially rich source of imaging biomarkers to inform DKD management [42]. Endothelial and microvessel dysfunction contribute to the development of DKD and premature cardiovascular disease amongst patients with diabetes [42]. Homology between the vasculature of the eye and the kidney suggests that inferences regarding the microvasculature of the kidney can be made from retinal imaging, providing a rationale to image accessible microvessels in the eye to improve DKD prognostication [42]. For example, retinal images were used to train and validate a deep learning algorithm which accurately predicted CKD status in community-based Asian cohorts [43]. The area under the receiver operating characteristic curve (AUC) of the deep learning algorithm improved when considered alongside conventional CKD risk factors such as age, gender, ethnicity, diabetes, and hypertension [43].

By capturing deeper vascular networks such as the choroidal circulation at near-histological resolution, the advent of optical coherence tomography (OCT) constitutes a major advance in retinal imaging which has transformed ophthalmology care [44]. OCT can now also be deployed in preclinical models of retinopathy [44]. Deep learning has been coupled with OCT imaging to triage and diagnose the commonest sight-threatening retinal diseases in an automated fashion and with similar accuracy to that of expert physicians [45]. Thus, combining the imaging power of cross-sectional chorioretinal OCT imaging with the analytical power of deep learning holds great promise as a means of developing prognostic imaging biomarkers related to adaptations of the renal microvasculature in people with diabetes mellitus [42].

### Histopathological and ultrastructural data

Similar to radiologic images of the kidney, digitised whole slide images (WSIs) and transmission electron microscopy (TEM) images of kidney biopsies contain a wealth of data which may be optimally analysed using deep learning approaches [46, 47]. Deep learning approaches may be used to automate the extraction of descriptive and quantitative structural features from WSIs and TEM images with improved reproducibility [46, 47]. The concept of reproducibility is an important one as although an inter-pathologist intra-class correlation coefficient (ICC) of 0.84 has been reported for assigning Tervaert glomerular class scores of DKD [48], there is still substantial room for improvement and ICC values are lower for other glomerular diseases such as IgA nephropathy and lupus nephritis [47]. Furthermore, semi- or wholly automated means of classifying DKD histologically would reduce personnel requirements and improve efficiency of assigning DKD diagnoses in routine clinical care.

A convolutional neural network architecture trained on PAS-stained kidney to segment six major renal structures (glomerular tuft, glomerulus including Bowman’s capsule, tubules, arteries, arterial lumina, and veins) demonstrated high performance in 5 murine disease models and the extracted features strongly correlated with data obtained from standard morphometric analysis [49]. Thus, deep learning may support high-throughput and reproducible quantitative feature extraction in experimental models of renal injury. Furthermore, the trained convolutional neural network performed well on human samples, thereby providing a link between automated histopathological assessment across the preclinical and clinical domains [49]. Indeed, a convolutional neural network was also used to segment PAS-stained kidney biopsy samples from 54 patients with DKD and classify them according to the Tervaert schema, achieving a high level of agreement with three independent pathologists [50]. The feasibility of deep learning-based segmentation of kidney WSIs has been demonstrated for multiple histologic stains including H&E, PAS, silver, and trichrome, with PAS-stained sections yielding the best concordance between pathologists and convolutional neural networks [51]. In the assessment of kidney structural features, deep learning has mainly been applied to digital pathology images thus far, although researchers have started to evaluate this strategy on TEM images with reasonable success [52].

## Molecular omics data

As highlighted in Figure 1, the increasing usage of a large number of omics modalities in DKD research has enriched understanding of disease pathogenesis, uncovered new means of patient stratification, and identified new treatment targets [15, 17, 16]. In particular, assessment of the genome, epigenome, transcriptome, proteome, metabolome, lipidome, and microbiome across *in vitro*, preclinical, and human studies has yielded consistent insights into DKD pathogenesis [15, 17, 16]. Some of the biomedical technologies which support omics analyses are outlined in Figure 1. Technological advances continue to be made, with the advent of scRNA-seq as well as spatially derived transcriptomics and metabolomics constituting some of the more recent notable examples with particular potential to uncover important mechanisms in the context of the vast cellular diversity of the kidney [53–55]. In many cases, the application of multiple technologies to characterise a particular molecular domain often provides complementary rather than redundant information.

Moreover, integration of data from several molecular domains is key to characterising the molecular heterogeneity of DKD, although integrative multi-omic analyses are not trivial owing to the complexity of the multiple high-dimensional datasets involved [17, 56]. It is also worth noting that changes in different molecular domains such as the transcriptome, the proteome, and the metabolome may not necessarily directly correlate [57, 58, 16]. For example, factors impacting translational efficiency will diminish mRNA-protein correlations for a given target, as will modalities of protein regulation other than gene transcription, such as post-translational modifications [57]. Furthermore, differences in the coverage of molecular domains by omics technologies may result in difficulties mapping insights from one to the other [58, 16]. For example, the low coverage of the metabolome (∼100-500 metabolites detected by existing technologies) compared with the high coverage of the transcriptome (∼20,000 genes measured by bulk RNA-seq) may limit attempts to infer relationships between transcripts and metabolites [58, 16]. Techniques that allow for integration of not only two data domains at a time (such as the transcriptome and the proteome) but also allow for simultaneous integration of clinical phenotypic data, imaging data, and histopathological data along with multiple molecular omics data domains are essential to gain more holistic insights into cellular function and interaction in a complex organ system such as the diabetic kidney [17, 56].

## Conclusions

The current one-size-fits-all approach to DKD care ignores the clinically apparent heterogeneity in disease prognosis and treatment-responsiveness [10]. It is hoped that a systems biology approach to DKD research will pave the way for a precision medicine approach to routine DKD care by unravelling individual-level molecular mechanisms which underlie progressive DKD and which are amenable to targeting by existing or novel therapeutic strategies [15, 16]. Certain priorities for translational DKD research which may be advanced by a systems nephrology approach include:

1. The development of model systems (*in vitro* or animal) which reliably recapitulate progressive and advanced human DKD characterised by single-cell and spatially resolved transcriptomics, thereby enhancing the translational relevance of preclinical DKD studies;
2. The identification of biomarkers which predict response to RAAS blockade, SGLT2is, and other emerging disease-modifying treatments for DKD in light of the inter-individual variability in treatment response; and
3. The delineation of mechanisms of DKD progression in the face of combined therapy with RAAS blockade and an SGLT2i, the current backbone of treatment, which may help to define targets for novel therapies which minimise the significant residual risk of progressive renal functional decline.

Integration of data from multiple model systems (*in vitro*, animal models, and patients) and from multiple domains (clinical phenotypic, imaging, histopathological/ultrastructural, and molecular omics) offers the best opportunity to realise the promise of systems nephrology in individualising DKD care. However, the efficacy of appropriately targeted novel therapeutics may still be impacted by inter-individual pharmacokinetic differences. Thus, pharmacogenomic profiling will play an important role in optimising outcomes for individuals with DKD.

Recent advances in the acquisition and integration of molecular omics data as well as enhanced understanding of machine/deep learning approaches offer renewed hope that the promise of systems nephrology for DKD care will be realised. While a comprehensive systems nephrology approach is now technically feasible in research studies, this must be balanced with plans for eventual implementation of elements of this paradigm in clinical practice. The value of biological insights derived from the refined techniques currently available must be balanced against their clinical translatability; researchers and clinicians alike should grapple with this compromise from the outset in an effort to prioritise which elements of the systems nephrology paradigm offer benefit to the largest number of patients in clinical practice. This will help to ensure that implementation of a systems nephrology approach in routine DKD care will not perpetuate, or indeed exacerbate, inequity in healthcare delivery.

## Figures and Tables

**Table 1:** An essential toolkit for a systems nephrology approach to diabetic kidney disease.^a^

Content	Rationale	Caveats
**Model systems**		
*In vitro*		
Kidney organoids	Understanding molecular mechanisms at single-cell resolution Amenable to genetic manipulation, such as CRISPR-Cas9 genome editing	Lack of integrated vasculature Not representative of mature nephron
Renal slice culture	Improved cellular composition and maturity compared with organoids	Less amenable to genetic manipulation than organoids
Animal models	Study disease at whole-tissue and whole-organism levels Cross-species comparison between humans and animals	No model recapitulates all features of human DKD, particularly advanced disease
Human consortia and biobanks (e.g., KPMP, TRIDENT, ENBiBA, BEAt-DKD)	Enhanced access to kidney tissue from patients with DKD with less inherent bias by minimising tissue obtained from clinically indicated biopsies	Must ensure diversity in populations recruited Data protection and privacy considerations
**Data sources/sample type**		
Clinical phenotypic data	Understanding of mechanisms and prognosis enhanced by data derived from routine clinical practice	Large studies require electronic health records or national/international registries Data protection and privacy considerations
Biofluids Blood Urine Faeces	Non-invasive, easily repeated over time Urine uniquely positioned to reflect molecular state of the kidney Faeces facilitates understanding of gut-kidney crosstalk mediated by the microbiome	May be considered surrogate/ancillary data sources rather than reflecting the direct molecular state of the kidney Requires agreement on standardised collection protocols
Kidney imaging	Non-invasive, easily repeated over time Simultaneously assess both kidneys and distinguish cortex from medulla	May require routine access to cross-sectional techniques such as magnetic resonance imaging Quantitative parameterisation can be machine-specific
Retinal imaging	Non-invasive, easily repeated over time Homology between vasculature of the eye and kidney Choroidal OCT permits granular assessment of deep vascular networks	May contribute redundant information alongside assessment of the microvasculature on kidney biopsy, although in theory could also obviate the need for kidney biopsy in certain circumstances
Kidney histopathology and ultrastructure	Directly reflects the structural state of the kidney Wealth of data may be extracted using deep learning approaches	Snapshot into disease progression to that point; serial assessment limited by invasive nature of the biopsy procedure Limited reproducibility, which may be improved by automated feature extraction
Molecular omics	Multiple molecular domains may be characterised in kidney tissue and biofluids offering complementary information Enriches understanding of disease and identifies new treatment targets Single-cell and spatially resolved modalities resolve the complex cellular composition of the kidney	Discovery studies are expensive and time-consuming Bias may be introduced at data acquisition and analysis stages, limiting reproducibility
**Computational methodologies**		
Data visualisation	Innovative approaches required to visually represent results from high-dimensional datasets which are difficult to summarise in text or tabular format	Overly complex visualisations may make the central message difficult to discern and discourage user interaction with the results
Multi-dimensional data (omics) integration	Further enhances understanding afforded by molecular omics analyses by identifying coherent signals between different molecular domains	Computationally demanding given the high-dimensional datasets inputted Limited by coverage of the molecular domains by different omics technologies
Machine and deep learning	Automated feature extraction in imaging and histopathological studies, improving efficiency/reproducibility while identifying features not visible to the human eye	Computationally demanding Deep learning’s black box may hinder understanding of why certain extracted features are of relevance
Clustering and multivariate outcome analyses	Classification of patient response according to molecular, imaging, histopathological, or other biomarkers	Model over-fitting may limit generalisability of results Multiple predictive variables from different domains may contribute redundant rather than complementary information

a BEAt-DKD, Biomarker Enterprise to Attack Diabetic Kidney Disease; CRISPR, clustered regularly interspaced short palindromic repeats; DKD, diabetic kidney disease; ENBiBA, European Nephrectomy Biobank; KPMP, Kidney Precision Medicine Project; OCT, optimal coherence tomography; TRIDENT, Transformative Research in Diabetic Nephropathy.
